# Supplementary material for: A Mixed Methods, Quasi-Experimental Evaluation Exploring the Impact of a Secondary School Universal Free School Meals Intervention Pilot
Source: Int J Environ Res Public Health. 2023 Mar 22;20(6):5216. doi: 10.3390/ijerph20065216 (PMC10049258; doi:10.3390/ijerph20065216)
Supplement: Supplementary file 1 [file ijerph-20-05216-s001.zip › ijerph-2213403-supplementary.pdf]

Bristol and Cardiff PHIRST

London Borough of Hammersmith and Fulham

Universal Free School Meals (UFSM) study

Student Survey

Front page:

This short questionnaire is part of a research study conducted by The University of Bristol.

Our study aims to understand what the impact of providing free school lunch has on the students, and their families.

The questionnaire should take no more than 15 minutes to complete.

- *I confirm that I have read the information letter dated xx/xx/*
- *I understand that my responses to this questionnaire will be shared with the research team at the University of Bristol.*
- *I understand that my responses will be put into the online repository, data.bris so they can be used for future research and learning. This means that other authorised researchers may use the survey responses in publications, reports, web pages, and other research outputs, as long as they have permission from the University of Bristol.*
- *I understand that my answers will be anonymous (no-one will know who I am from my answers).*
- *I understand and agree that the answers I give in the survey may be used when the research findings are shared with others, for example in publications, reports, and web pages. I understand that I will not be named when the findings are shared.*
- *I understand that I can choose not to take part, or to stop answering the survey questions at any time.*

If the respondent answers 'NO' to any of the above, they will see the following message.

Thank you. Your responses indicate that you do not give informed consent to take part in the survey.

If the respondent answers 'YES' to all of the above, they will be directed to the next page of the questionnaire.

Thank you.

The following questions are things about you:

1. What year group are you in?
2. Are you a boy/girl/prefer to self-describe/prefer not to say.

3. What best describes your ethnic group? Please pick one of the following:

White

1. English / Welsh / Scottish / Northern Irish / British

2. Irish

3. Gypsy or Irish Traveller

4. Any other White background, please describe

Mixed / Multiple ethnic groups

5. White and Black Caribbean

6. White and Black African

7. White and Asian

8. Any other Mixed / Multiple ethnic background, please describe

Asian / Asian British

9. Indian

10. Pakistani

11. Bangladeshi

12. Chinese

13. Any other Asian background, please describe

Black / African / Caribbean / Black British

14. African

15. Caribbean

16. Any other Black / African / Caribbean background, please describe Other ethnic group

17. Arab

18. Any other ethnic group, please describe

Thank you.

The following questions are about the food situation in your home **during the last month**. Please choose the answer that best describes you.

4. Did you **worry** that food at home would run out before your family got money to buy more?

_______ A LOT

_______ SOMETIMES

_______ NEVER

5. Did the food that your family bought **run out**, and you didn't have money to get more?

______ A LOT

______ SOMETIMES

______ NEVER

6. Did your meals only include a few kinds of **cheap foods** because your family was running out of money to buy food?

______ A LOT

______ SOMETIMES

______ NEVER

7. How often were you not able to eat a **balanced meal** because your family didn't have enough money?

______ A LOT

______ SOMETIMES

______ NEVER

8. Did you have to **eat less** because your family didn't have enough money to buy food?

______ A LOT

______ SOMETIMES

______ NEVER

9. Has the size of your meals **been cut** because your family didn't have enough money for food?

______ A LOT

______ SOMETIMES

______ NEVER

10. Did you have to **skip a meal** because your family didn't have enough money for food?

______ A LOT

______ SOMETIMES

______ NEVER

11. Were you **hungry** but didn't eat because your family didn't have enough food?

    _____ A LOT

    _____ SOMETIMES

    _____ NEVER

12. Did you not eat for a **whole day** because your family didn't have enough money for food?

    _____ A LOT

    _____ SOMETIMES

    _____ NEVER

Thank you.

The next few questions are about the foods that you eat.

Please tell us how often you eat the following items:

14. crisps

("never"/"less than once a week"/"once or twice a week"/"most days"/"once a day"/"more than once a day").

15. sweets/chocolate

("never"/"less than once a week"/"once or twice a week"/"most days"/"once a day"/"more than once a day").

16. cakes

("never"/"less than once a week"/"once or twice a week"/"most days"/"once a day"/"more than once a day").

17. other puddings/deserts

("never"/"less than once a week"/"once or twice a week"/"most days"/"once a day"/"more than once a day").

18. biscuits.

("never"/"less than once a week"/"once or twice a week"/"most days"/"once a day"/"more than once a day").

19. chips

(“never”/“less than once a week”/“once or twice a week”/“most days”/“once a day”/“more than once a day”).

20. sausages/burgers

(“never”/“less than once a week”/“once or twice a week”/“most days”/“once a day”/“more than once a day”).

21. Sugary or fizzy drinks

(“never”/“less than once a week”/“once or twice a week”/“most days”/“once a day”/“more than once a day”).

Thank you, you have completed the questionnaire. Please remember your answers are anonymous and no-one will know what you share.
